# Financing and cost-effectiveness analysis of public-private partnerships: provision of tuberculosis treatment in South Africa

**DOI:** 10.1186/1478-7547-4-11

**Published:** 2006-06-06

**Authors:** Edina Sinanovic, Lilani Kumaranayake

**Affiliations:** 1 Health Economics Unit, School of Public Health & Family Medicine, Faculty of Health Sciences, University of Cape Town, Anzio Rd, 7925 Observatory, South Africa; 2 Department of Public Health and Policy, London School of Hygiene and Tropical Medicine. Keppel St., London, WC1E 7HT, UK

## Abstract

**Background:**

Public-private partnerships (PPP) could be effective in scaling up services. We estimated cost and cost-effectiveness of different PPP arrangements in the provision of tuberculosis (TB) treatment, and the financing required for the different models from the perspective of the provincial TB programme, provider, and the patient.

**Methods:**

Two different models of TB provider partnerships are evaluated, relative to sole public provision: public-private workplace (PWP) and public-private non-government (PNP). Cost and effectiveness data were collected at six sites providing directly observed treatment (DOT). Effectiveness for a 12-month cohort of new sputum positive patients was measured using cure and treatment success rates. Provider and patient costs were estimated, and analysed according to sources of financing. Cost-effectiveness is estimated from the perspective of the provider, patient and society in terms of the cost per TB case cured and cost per case successfully treated.

**Results:**

Cost per case cured was significantly lower in PNP (US $354–446), and comparable between PWP (US $788–979) and public sites (US $700–1000). PPP models could significantly reduce costs to the patient by 64–100%. Relative to pure public sector provision and financing, expansion of PPPs could reduce government financing required per TB patient treated from $609–690 to $130–139 in PNP and $36–46 in PWP.

**Conclusion:**

There is a strong economic case for expanding PPP in TB treatment and potentially for other types of health services. Where PPPs are tailored to target groups and supported by the public sector, scaling up of effective services could occur at much lower cost than solely relying on public sector models.

## Background

In line with the global tuberculosis-related Millennium Development Goals to halt and begin to reverse the incidence of tuberculosis (TB) by 2015, the Stop TB Partnership set targets to halve TB prevalence and death by 2015 [1]. To achieve and sustain these targets, a variety of provision and financing models needs to be considered in developing country health systems. Even with strengthened public health provision, the resulting outcomes may not be as expected. Recent analysis of tuberculosis (TB) control goals found that despite broad coverage through public programmes, proportionate increases in case-detection rates were not achieved. Projections suggest that at current rates, even if there were 100% global coverage by DOTS – the internationally recommended control strategy for TB, only half of new infectious cases would be detected [2]. Key reasons for this are that patients do not have access to public health facilities or seek care from providers not linked to national programmes or the public health system – such as private doctors working in their own facilities or for employer health services [2,3]. Private TB treatment provision has been characterised by limited notification of cases from the private sector, inappropriate drug regimens, and unknown treatment outcomes [4,5].

Public sector collaboration with private providers can range from provision of information and education to formal public-private partnerships (PPPs) with small-scale contracting of service components or larger-scale sharing of health care provision and financing [6-9]. PPPs seek to complement rather than substitute for public health services. Partnerships with traditional healers, community-based organisations and private practitioners can provide a number of benefits, including increased numbers of people receiving services, leveraging of additional resources, improved private provider technical capacity, and adherence to national protocols helping to minimise drug resistance [8].

South Africa is one of several countries where partnerships between the public and private sectors have been recognised as a policy objective [10], with the National Treasury developing its own guidelines for PPPs related to design, procurement and implementation [11]. In response to the dual TB/HIV epidemic, the national tuberculosis programme has began to collaborate with different private providers in the provision of TB treatment. This study explores cost and cost-effectiveness of different existing PPP arrangements for TB DOTS provision. The financing required for the different models from the perspective of the provincial TB programme, provider, and the patient are also estimated. Previous economic studies have shown a reduction in costs and improvement in cost-effectiveness of DOTS through decentralisation and strengthened community-based care [12-18]. Besides the study on the cost-effectiveness of two public-private mix pilot projects in India [19] to-date there have been no published studies on the cost-effectiveness of PPP involving private providers for the purposes of TB diagnosis and treatment, nor studies on the public sector financing requirements for any type of PPP – the latter is critical if considering the potential scale-up or replication of PPPs.

## Methods

### Background

The South African National TB Control Programme adopted the DOTS strategy in 1996. South Africa has achieved the WHO target rate of 70% case detection, but despite DOTS population coverage of 93% in 2004, the treatment success rate of 67% [20] remains well below the WHO target of 85%. The TB epidemic is fuelled by the HIV epidemic with over 6.5 million people infected with HIV in South Africa in 2002 [21], and 60% of TB adult patients also HIV positive [20].

### Models of provision

Three different models of DOT provision were evaluated for different target groups: purely public, public-private workplace partnership (PWP), and public-non-governmental organisation partnership (PNP). The justification for using three models as case studies was that they complement each other by demonstrating a range of models of provision, all of which may be relevant in particular settings. In the public model, patients are diagnosed and treated in public clinics, with direct observation undertaken in the public health facilities by health workers following national treatment guidelines [22]. The PWP model represents a partnership between provincial TB programmes and mining companies where the employer's occupational health services are either reimbursed per patient day or receive free drugs for each TB patient treated in their clinics. The type of reimbursement depends on the provincial regulations. Mining companies were selected, as there is a long history of TB treatment in the industry. The PNP model is a partnership between provincial TB programmes and non-governmental organisations (NGOs) providing community-based DOT in which these NGOs are paid a monthly sum per patient to manage community-based TB programmes. In this model, patients are diagnosed and monitored in public clinics for the first 10 days. Subsequent treatment is directly observed by community health worker 'treatment supporters' in the community. In addition to the funding from the provincial TB control programme, these NGOs receive funding from other sources such as charities and donors. In return for payments from provincial TB programmes both private partners are required to follow national treatment guidelines, complete and submit standardised quarterly reports to district TB coordinators, and liaise with district public health facilities.

### Site selection

For each type of provision model, 2 sites were selected (Table 1). Sites were identified using key informant interviews with provincial TB programme officials, and then selected by purposive sampling according to their availability, urban-rural locations, different reimbursement mechanisms, and willingness to participate in the study. Differentials among sites in terms of TB incidence and HIV prevalence reflected the variation in target populations reached by the PPPs.

**Table 1:** Key characteristics of each site

**Model/Site**	**PWP model**		**PNP model**		**Purely public model**	
	
	Site 1 (N = 95)	Site 2 (N = 423)	Site 3 (N = 355)	Site 4 (N = 50)	Site 5 (N = 85)	Site 6 (N = 174)
Type of provision	Private workplace	Private workplace	Private non-governmental	Private non-governmental	Public	Public
Type of facility	Occupational health clinic	Occupational health clinic	Clinic working closely with a local NGO	Clinic working closely with a local NGO	Health clinic	Health clinic
Location (Province)	Near large rural town in North West	Near small rural town in Free State	Urban informal settlement in Western Cape	Rural informal settlement in Western Cape	Small rural town in Western Cape	Urban city area in Western Cape
Population served	Low income workers, predominantly male	Low income workers, predominantly male	Low income residents, male and female adults, high unemployment	Low income residents, male and female adults, high unemployment	Low income residents, male and female adults, high unemployment	Low income residents, male and female adults, high unemployment
TB incidence per 100 000 population*	1 073	3 012	439	149	169	176
Approximated HIV prevalence in the study population**	(*approx*) *44%*	(*approx*) *48%*	(*approx*) *39%*	(*approx*) *36%*	(*approx*) *29%*	(*approx*) *23%*
Overall TB service range^†^	Surveillance for TB, diagnosis and treatment	Surveillance for TB, diagnosis and treatment	Diagnosis, treatment, and social support	Diagnosis, treatment, and social support	Diagnosis and treatment	Diagnosis and treatment
Case finding	Annual radiological screening; passive, and contact tracing	Annual radiological screening; passive, and contact tracing	Passive	Passive	Passive	Passive
Diagnosis	Sputum smears; all patients with suspected pulmonary TB should have 1 sputum specimen submitted for culture	Sputum smears; all patients with suspected pulmonary TB should have 1 sputum specimen submitted for culture	Sputum smears; 1 sputum for culture if smear negative at diagnosis and unresponsive to a course of antibiotics	Sputum smears; 1 sputum for culture if smear negative at diagnosis and unresponsive to a course of antibiotics	Sputum smears; 1 sputum for culture if smear negative at diagnosis and unresponsive to a course of antibiotics	Sputum smears; 1 sputum for culture if smear negative at diagnosis and unresponsive to a course of antibiotics
DOT system in place	Hospitalisation for the first 7 days followed by DOT by nurses in the occupational clinics	DOT by nurses in the occupational health clinics	DOT by nurses in the public clinic for the first 10 days followed by DOT by 'treatment supporters' in the community	DOT by nurses in the public clinic for the first 10 days followed by DOT by 'treatment supporters' in the community	DOT by nurses in the public clinic	DOT by nurses in the public clinic

* Source for TB prevalence: providers' annual reports.

** Approximated by the clinic staff as no specific prevalence studies undertaken in clinic target populations. One of the reasons for higher HIV prevalence in sites 1 and 2 could be attributed to better case detection and follow-up in the PWP sites.

^† ^Due to resource constraints, retrieval of defaulters is rarely done in the public sector. In the PNP model, if a patient does not attend, treatment supporters are expected to visit the patient's home within 24 hours and to report this to the public clinic.

In the PWP model, compliance rate is extremely high mainly because of the system of 'parading' (a patient is not allowed to work if defaulting) which is in place in the mining companies where a patient has no choice but adhere to the treatment.

### Procedure

Data collection was undertaken between March 2002 and February 2003. Written consent was obtained from all the study providers. A verbal patient consent was also sought. Ethical approval was obtained from both the University of Cape Town and London School of Hygiene and Tropical Medicine ethical committees.

### Cost and cost-effectiveness

The cost analysis was retrospectively undertaken from a provider perspective and a societal perspective. Costs were collected for a 12-month period in each facility between 2000 and 2001. Average, rather than marginal, economic costs were assessed, which are more relevant for national policy [23]. An ingredients-based costing methodology was used where quantities of resources were multiplied by their respective prices to obtain total costs. Capital costs were annualised using life expectancies of 30 years for buildings, 10 years for equipment, 5 years for vehicles and training, and the standard discount rate of 3% [24].

Methods used for measuring, identifying and valuing the costs of TB treatment are given in Table 2. The average cost of managing a new smear-positive patient from diagnosis to completion of treatment for each site was calculated by multiplying the average cost of each treatment component by the number of times the cost was incurred according to the site-specific clinic protocols.

**Table 2:** Methods used for measuring, identifying and valuing the costs of TB treatment

**Type of cost**	**Identification**	**Measurement**		**Valuation**	
	
	Categories	Costing method	Sources of data	Valuation method	Sources of data
**Recurrent**					
Personnel	Administration and management, clinical staff (doctors, nurses, lay workers*), and support staff (cleaning)	Percentage of time spent on different activities	Observation** and semi-structured interviews with providers	Total remuneration package costs	Provider expenditure reports
Supplies	Sputum and culture tests, x-rays and drugs	Quantity consumed	Patient records	Market prices	Provincial and private laboratories and pharmacy price lists
Vehicle operating and maintenance	Vehicle running costs	Number of kilometres travelled	Vehicle logbook, interview with clinic manager	Actual expenditure on fuel, oil and maintenance	Automobile Association rates
Building operating and maintenance	Overheads (water, electricity, telephone, fax, stationeries etc)	Proportion of total visits for which TB patients accounted	Actual costs from facility records, interview with clinic manager	Actual expenditure	Provider expenditure reports

**Capital**^†^					
Buildings	Offices, clinics and hospitals	Proportion of total visits for which TB patients accounted	Interview with clinic manager	Replacement prices	CSIR Building and Construction Technology
Equipment	Furniture, medical and non-medical equipment	Proportion of total visits for which TB patients accounted)	Interview with clinic manager	Replacement prices	Local manufacturers
Vehicles	Vehicles used for TB patients	Vehicle utilisation (km travelled)	Vehicle log book, interview with clinic manager	Replacement prices	Local car dealers
Training	Community "treatment supporters" training	Number of treatment supporters trained	Actual costs from NGO records	Actual expenditure	Provider expenditure reports

* Average cost 'treatment supporter' visits was based on the NGO payment per visit.

** Observation was used to determine clinic staff time spent on each type of visit made by adults. No variations in terms of the HIV status and gender of the patient were done.

^† ^Life expectancies of buildings were 30 years, equipment 10 years, vehicles and training 5 years, and the standard discount rate of 3% (*24*).

Patient costs (time and travel costs) were estimated using a retrospective structured patient interview. A sample of 20 randomly chosen patients in each site was used due to time constraints. No patients were interviewed in the PWP model as DOT is provided in the workplace. Patient costs of seeking care prior to the facility where they were interviewed were excluded from the analysis. Time costs were converted to a monetary value based on the average income reported by the patients interviewed [24]. Diagnosis and treatment of TB are free of charge to patients in all study sites.

To calculate the effectiveness measures, the cohort of all new smear-positive TB patients who started treatment during the 12-month costing period was followed to obtain the treatment outcomes. New smear-positive cases were selected because they are the most infectious cases, and therefore have the highest public health importance; the South African DOTS strategy focuses primarily on improving the cure rate among new patients; and data on these cases were readily obtainable. The cost-effectiveness ratio was calculated for each model of provision by dividing cost by the unit of effect and compared with each other. As these partnership models use resources from three different sources (provider, patient and government), both a provider and societal perspective is adopted for the cost-effectiveness analysis.

### Sensitivity analyses

To reflect the uncertainty inherent in the analysis, four univariate sensitivity analyses were performed: using alternative assumptions regarding provider costs (reduction of 50% of value of staff time in baseline analysis due to a possibility that staff time could have been overestimated); using alternative assumptions regarding patient costs (limits of 95% confidence interval); variation in discount rates (3% below and above the rate used in baseline analysis); and adjusting effectiveness data by the death rate in order to explore the importance of different HIV prevalence across sites. Effectiveness was recalculated by adjusting the total number of patients in the cohort by the total number of deaths in the same cohort. Different assumptions about provider and patient costs were examined because they constitute the major costs. A multivariate sensitivity analysis, where estimates of provider cost and effectiveness adjusted by the death rate are varied at the same time, was also performed.

## Results

### Average cost per patient treated

The cost per TB patient treated varied across the models of treatment provision (Table 3), with the highest costs for the PWP models (US$ 654–744) reflecting different protocols related to hospitalisation (site 1) and procurement of drugs. In site 2, the only site without public financing of drugs, 52% of the entire treatment cost was drugs. The PNP model had the lowest cost per patient treated and was less than half of that found in the PWP model. DOT was the most significant cost in public clinics (84–85%) and 40–51% of costs in PWP sites. The largest cost component for PNP was the overall supervision of the programme (28–29%).

**Table 3:** Average provider costs (% of total), mean patient costs (95% confidence interval) and financing (% of total), US$*

**Average provider costs**	**PWP model**		**PNP model**		**Purely public model**	
	
	Site 1	Site 2	Site 3	Site 4	Site 5	Site 6
	N = 95	N = 423	N = 355	N = 50	N = 85	N = 174
Hospital stay**	220 (34%)	N.A.^††^	N.A.	N.A.	N.A.	N.A.
Health clinic visits for monitoring**	14 (2.1%)	18 (2.4%)	23 (9%)	24 (9%)	19 (4%)	20 (3%)
Health clinic visits for DOT**	334 (51%)	301 (40%)	36 (14%)	39 (16%)	426 (84%)	485 (85%)
Visits to 'treatment supporters'**	N.A.	N.A.	55 (22%)	55 (22%)	N.A.	N.A.
Sputum smears^†^	12 (1.8%)	14 (1.8%)	16 (7%)	16 (7%)	16 (3%)	16 (3%)
Sputum culture^†^	16 (2.4%)	17 (2.3%)	N.A.	N.A.	N.A.	N.A.
Drugs	46 (7%)	383 (52%)	46 (18%)	46 (18%)	46 (9%)	46 (8%)
X-rays^†^	12 (1.8%)	11 (1.5%)	N.A.	N.A.	N.A.	N.A.
Overall supervision of community-based programme	N.A.	N.A.	74 (29%)	72 (28%)	N.A.	N.A.
Training for community 'treatment supporters'	N.A.	N.A.	1 (0.4%)	1 (0.4%)	N.A.	N.A.
Total cost per patient	654	744	251	253	507	568

**Mean patient cost**						
Visits to clinic for monitoring and DOT						
*Travel cost*	N/A	N/A	2.1 (1.7–2.4)	2.6 (2.3–3.2)	26.2 (25.1–27.6)	20.3 (18.6–23.8)
*Time cost*	N/A	N/A	6.2 (4.0–8.3)	5.9 (4.6–6.5)	75.6 (71.8–78.2)	101.9 (94.4–115.1)
Visits to 'treatment supporter' for DOT						
*Travel cost*	N/A	N/A	0	0	N.A.	N.A.
*Time cost*	N/A	N/A	30.9 (23.6–36.8)	28.2 (24.9–30.5)	N.A.	N.A.
Total cost per patient	N/A	N/A	39.2 (28.3–47.5)	36.7 (31.8–40.2)	101.8 (96.9–105.8)	122.2 (113.0–138.9)

**Financing of treatment cost**						
Public provider	N.A.	N.A.	59 (20%)	63 (20%)	445 (73%)	506 (73%)
Private provider	609 (93%)	708 (95%)	121 (42%)	114 (39%)	N.A.	N.A.
Provincial TB programme^‡^	46 (7%)	36 (5%)	71 (24%)	76 (27%)	62 (10%)	62 (9%)
Patient	N.A.	N.A.	39 (13%)	37 (13%)	102 (17%)	122 (18%)
Total cost per patient	654	744	290	290	609	690

* Cost data from 2001. Average exchange rate prevailing in 2001 US$1 = R8.57.

** Expected number of visits/hospital days for each site is as follows: 7 hospital days for site 1; 3 visits for monitoring at each site except at site 1 where there are only 2 such visits; 130 visits for DOT (sites 2, 5 and 6), 123 visits for DOT (site 1), and 10 visits for DOT (sites 3 and 4); 120 visits to 'treatment supporter' (sites 3 and 4).

^† ^Expected number of diagnostic tests for each site: 4 sputum smears (site 1), 7 sputum smears (site 2), and 4 sputum smears (sites 4–6); 1 sputum culture (sites 1 and 2); 3 X-rays (sites 1 and 2).

^†† ^N.A. = not applicable.

^‡ ^Provincial TB programme covered the cost of drugs in all the sites except in site 2 where a reimbursement on a patient day basis was provided. In addition to the drugs, the programme also covered the cost of diagnostic tests in sites 3–6.

The cost to the patient in the purely public model was between 2.6 and 3.3 times higher than the PNP model reflecting less time that a patient had to spend getting the treatment in the community and lower transport cost (Table 3).

The costs of TB treatment were largely financed by the main provider in each site (Table 3). PWP providers financed 93–95% of the total cost with a small 5–7% contribution required from the provincial TB programme. In contrast, the provincial TB programme contributed 24–27% of financing towards PNP sites, with the public sector also paying for another 20% of costs. However, in budgetary terms this meant that the provincial TB programme contributed only up to twice the amount of funding to PNP sites as it did for PWP, reflecting the much lower cost per patient treated ($290) in the PNP sites versus the public or PWP sites.

### Treatment outcomes

Table 4 shows treatment outcomes for all new smear-positive cases in the cohort. The treatment success rate ranged between 74% (site 6) and 89% (site 5) and the cure rate ranged between 65% (site 3) and 87% (site 5). The major influence on effectiveness were high mortality rates of 12% and 11% in sites 1 and 2 respectively, and high defaulting rates of 24% and 16% (and so lower cure rates) in sites 3 and 6 respectively which were the only 2 urban sites. No one model performed unambiguously better than the others.

**Table 4:** Treatment outcome and cost-effectiveness (CE) for each model of treatment provision in 2001 US$*

Treatment outcome**	**PWP model**		**PNP model**		**Purely public model**	
	
	Site 1	Site 2	Site 3	Site 4	Site 5	Site 6
	
	N = 95	N = 423	N = 355	N = 50	N = 85	N = 174
Successfully treated^†^	83 (87%)	368 (87%)	283 (80%)	41 (82%)	76 (89%)	129 (74%)
Cured^††^	79 (83%)	321 (76%)	231 (65%)	41 (82%)	74 (87%)	121 (69%)
Failed^‡^	1 (1%)	4 (1%)	3 (1%)	1 (2%)	0	1 (1%)
Died^‡‡^	11 (12%)	47 (12%)	13 (4%)	4 (8%)	1 (1%)	4 (2%)
Interrupted^§^	-	-	56 (15%)	4 (8%)	8 (10%)	40 (23%)

**CE from the provider perspective**						
Total cost of treating patient	654	744	251	253	507	568
Treatment success rate^§§^	87	87	80	82	89	74
Cure rate^∥^	83	76	65	82	87	69
Cost per new smear-positive patient successfully treated	752	855	314	308	570	767
Cost per new smear-positive patient cured	788	979	386	308	583	823

**CE from the patient perspective**						
Total cost of treating patient	N.A.	N.A.	39	37	102	122
Treatment success rate			80	82	89	74
Cure rate			65	82	87	69
Cost per new smear-positive patient successfully treated			49	45	115	165
Cost per new smear-positive patient cured			60	45	117	177

**CE from the societal perspective**						
Total cost of treating patient^∥ ∥^	654	744	290	290	609	690
Treatment success rate	87	87	80	82	89	74
Cure rate	83	76	65	82	87	69
Cost per new smear-positive patient successfully treated	752	855	362	354	684	932
Cost per new smear-positive patient cured	788	979	446	354	700	1 000

* Cost data from 2001. Average exchange rate prevailing in 2001 US$1 = R8.57.

** Source: Reports submitted to the provincial TB programmes.

^† ^The sum of those patients who were cured plus those who completed treatment but without laboratory proof of cure.

^†† ^Patients who were smear negative at the end of treatment.

^‡ ^Patients who remained or become again smear-positive at 5 months or later during treatment. Patients who were 'transferred out' are not included in the denominator (to be in line with international and national reporting requirements, this category of TB patients will in future be included in the denominator of the reporting district where they were initially registered). The number of patients who were 'transferred out': 1 (site 1), 4 (site 2), 31 (site 3), 9 (site 4), 16 (site 5) and 35 (site 6).

^‡‡ ^Patients who died for any reason during the course of TB treatment.

^§ ^Patients whose treatment was interrupted for 2 months or more.

^§§ ^Estimated as a ratio between the number of new smear-positive patients registered and the number of new smear-positive patients successfully treated.

^∥ ^Estimated as a ratio between the number of new smear-positive patients registered and the number of new smear-positive patients cured.

^∥ ∥^This represents a sum of provider and patient costs for each site.

### Cost-effectiveness

A provider-only perspective shows the most cost-effective TB treatment was the PNP model reflecting both lower cost per patient treated and relatively higher treatment success and cure rates (Table 4). The least cost-effective TB treatment was the PWP model, where sites achieved relatively higher successful treatment success and cure rates, but the average costs per patient treated were the highest.

From the patient's perspective, the most cost-effective TB treatment was the PWP model (TB treatment was workplace-based and the employer covered the cost of transport), followed by the PNP model (TB treatment was community-based and accessible to the patient). The least cost-effective was the purely public model, reflecting long waiting hours and poor geographical accessibility of public clinics. From the social perspective, the PNP model remained the most cost-effective model of all the models of provision. However, from a societal perspective, there were similar ranges for cost-effectiveness between the PWP model and the purely public model.

### Sensitivity analyses

The results were robust in sensitivity analyses (Table 5) with cost and cost-effectiveness assumptions most sensitive to valuation of staff time. The relative rankings remained the same. Sensitivity analysis using the number of registered patients adjusted by the number of the patients who died during the treatment showed that results were not sensitive to plausible variability in the effectiveness data. Cost-effectiveness in the higher HIV prevalence sites improved considerably when compared to other study sites.

**Table 5:** Sensitivity analyses

	**PWP model**		**PNP model**		**Purely public model**	
	
	Site 1	Site 2	Site 3	Site 4	Site 5	Site6
	
**Alternative estimates of provider costs (staff time)**						
• % divergence from base-case provider cost estimate	-15%	-13%	-8%	-8%	-24%	-28%
• % divergence from base-case provider cost-effectiveness estimate	-15%	-13%	-8%	-8%	-24%	-28%
**Lower limit patient cost**						
• % divergence from base-case patient cost estimate	N.A.*	N.A.	-5%	-5%	-5%	-5%
• % divergence from base-case patient cost-effectiveness estimate	N.A.	N.A.	-4%	-3%	-1%	-1%
**Upper limit patient cost**						
• % divergence from base-case patient cost estimate	N.A.	N.A.	+5%	+5%	+5%	+5%
• % divergence from base-case patient cost-effectiveness estimate	N.A.	N.A	+3%	+0.2%	+0.6%	+2%
**0% discount rate**						
• % divergence from base-case provider cost estimate	-3%	-3%	-3%	-3%	-3%	-3%
• % divergence from provider cost-effectiveness estimate	-2%	-0.6%	-0.5%	-2%	-1%	-0.5%
**6% discount rate**						
• % divergence from base-case provider cost estimate	+3%	+3%	+3%	+3%	+3%	+3%
• % divergence from provider cost-effectiveness estimate	+3%	+2%	+2%	+3%	+2%	+2%
Effectiveness adjusted by the death rate**						
• % divergence from base-case treatment success rate	-12%	-11%	-4%	-8%	-1%	-2%
• % divergence from base-case societal cost-effectiveness estimate (treatment success rate)	-12%	-11%	-4%	-8%	-1.6%	-2.5%
• % divergence from base-case cure rate	-12%	-11%	-4%	-8%	-1%	-2%
• % divergence from base-case societal cost-effectiveness estimate (cure rate)	-12%	-11%	-4%	-8%	-1%	-2%
**Multivariate analysis (estimates of provider cost and effectiveness adjusted by the death rate are varied at the same time)**						
• % divergence from base-case provider cost estimate	-15%	-13%	-8%	-8%	-24%	-28%
• % divergence from base-case cure rate	-12%	-11%	-4%	-8%	-1%	-2%
• % divergence from base-case societal cost-effectiveness estimate	-25%	-23%	-11%	-15%	-25%	-30%

* N.A. = not applicable.

**Effectiveness was re-calculated by adjusting the total number of patients in the cohort by the total number of deaths in the same cohort. In this case, the treatment success rate = [number of successfully treated patients/(total number of patients – number of patients who died during treatment)] * 100. For example, in site 1, the treatment success rate adjusted by the death rate = [83/(95 - 11)] * 100 = 99%.

## Discussion

This study provides evidence on the cost-effectiveness of different PPP models for the provision of TB treatment in South Africa. In the two PPP models, increased community involvement and availability of treatment at a workplace were more affordable to the public sector. The effectiveness was also better in the PPP models, suggesting that the public-private models of provision are more effective than the purely public model. This may be due to superior quality of care provided in public-private settings [25]. Overall, the PNP model was consistently the most cost-effective model, similar to results elsewhere evaluating community-based models [13-19]. However, these results are preliminary due to a limited number of sites available for each model and therefore cannot provide definite conclusions. The study is context-specific and exploratory in its nature as it evaluates exiting models and does not provide any definite results.

The new costs associated with the community-based treatment to the public sector were small in comparison with the cost of DOT in public clinics. The incentive paid to community treatment supporters (on average US$ 0.3 per visit) was much lower than the average cost of a clinic visit (on average US$ 3.5 per visit). The availability of the treatment at a workplace and in the community resulted in substantial savings to the patient. In addition to the cost reduction to the public sector, by providing TB treatment to poorer community members and employees, the treatment became more accessible and convenient for patients in both study populations reducing costs to the patient by 64%–100%.

It is also important to consider where the employer-based and community-based approaches are of greatest relevance. Arguably, the employer-based TB treatment is most appropriate in companies which employ a large number of people, where occupational health clinics exist and where TB is an occupational health problem, as it is in the mining industry. In the case of the community-based TB treatment, it is most appropriate in areas where public sector clinics are already working at capacity; where clinic-based care is not achieving high cure rates; where geographical access to health facilities for patients is poor; and where more affordable but still cost-effective approaches are required due to health service budget cuts and/or increases in the number of TB cases. The different ways in which community-based TB treatment can be implemented depend on the level of socio-economic development, the degree of social mobilisation for TB among other health activities, and the particular cultural setting (Maher et al 1999). While the principles of community contribution of TB are generalisable (e.g. close links between the general health services, TB control programme and community-based NGOs), the details of how this model of service delivery is designed and implemented will depend largely on the specific setting.

The study has a number of limitations. Non-randomised purposive sampling was used to identify sites that reflected communities and locations where PPPs are currently in place. Thus the sites varied significantly in terms of TB incidence due to the nature of the PPP target groups. Although efforts were made to try and select sites with similar HIV prevalence rates, the nature of the PPP target groups and scarcity of data on HIV prevalence made this difficult. As there is a higher mortality rate for HIV+ TB patients, we were not able to control for the fact that TB treatment outcomes might have been poorer in sites with a higher HIV prevalence rate than in sites with a lower HIV prevalence rate. This could underestimate the cost-effectiveness at higher HIV/TB incidence sites. However, it is difficult to discuss the importance of HIV prevalence differences on the study results as HIV prevalence rates are based on estimates by the clinic staff. The study results might also be different if smear negative cases were selected. The PPPs were all relatively well functioning (with cure rates well above the South African average of 55%). The purposive sampling may have led to identification of better-functioning PPPs, which may suggest that results are optimistic. The conventional practice of using average reported incomes among a sample of patients is used to value patient time. This might have under or over-estimated true costs. Finally, whilst important, the time cost of identifying, selecting, initiating and sustaining different PPPs, and the "stewardship" costs, such as ensuring that the private partners follow TB treatment guidelines, to government were not included in the analysis. We estimate these costs to be around US $35,000 per year per province (this is the salary cost of a middle-level manager whose main responsibility would be to manage PPPs for the provision of TB). In addition, a range of incentives should be used to encourage private sector participation in partnership with the government for the provision of TB treatment. While recognising that 'getting incentives right' is important in any principal-agent relationship, monitoring and evaluation of the partnerships, however, may not always be easy and other factors, such as levels of trust and perceptions of relative risks, may potentially play an essential role in underpinning efficient public-private relationships.

## Conclusion

Overall, the results suggest there is a strong economic case for expanding PPP involvement in TB treatment in the process of scaling up. The cost per new patient treated to government could be reduced by enhanced partnership between the private and public sectors. Expansion of PPPs could potentially lead to reduced government sector financing requirements for new patients: government financing would require $609–690 per new patient treated, in contrast to PNP sites which would only need to $130–139 per patient (almost a five-fold reduction in costs), and $36–46 (a fifteen fold reduction) with the PWP model. The study models are comparable in that they follow the same TB treatment protocol, are similar in terms of key social, economic and demographic characteristics, and provide care to the lower-income populations. The major caveat to using the study results in making policy recommendations is that it was based on a small number of study sites. Whilst including such a small sample of sites should not have influenced losing any rigour, the results should be interpreted as preliminary.

Tuberculosis treatment provided through the PPPs also seems to be more accessible and convenient for patients in both study populations. Through partnership with the government, mining companies and community-based NGOs can take most of the responsibility for their own employees and associated communities respectively. The PWP model appears to be more sustainable than the PNP model, mainly due to dependency of the NGOs on external financing. Moreover, employers have an economic incentive to keep the workforce healthy.

This study evaluated different models of provision for different study populations. Ideally, an experiment should be set up to compare different models for the same population. In urban areas, both PNP and public sites had greater default rates. This may well improve by using PPPs involving other types of private providers such as private practitioners in urban areas. Currently these types of arrangements are limited relative to other parts of the world [26-28].

Expansion may require increased investment in the PPPs, but they seem to be capable of delivering important improvements in the affordability and efficiency of TB treatment, and improving the South African health system's capacity to cope with the impact of the HIV/AIDS epidemic. Regardless of the model, the provincial TB programmes had to finance some proportion of the total cost per patient (although almost 50% lower for PWP models). Managing PPPs, however, is challenging as they embody a complex set of relationships between public and private actors and require careful monitoring [8]. These findings could also be applicable to PPPs in the provision of other services such STI treatment [29] and reproductive health services [30]. PPPs should be seen as complementary to public services as the different models serve different target groups. These results show that where PPPs are tailored to the context and well-supported by the public sector, scaling-up of effective services could occur at much lower cost relative to solely scaling up traditional public sector models. However, an assessment of the resources required from the government for training, monitoring and quality control of PPPs is crucial, and further research into financial sustainability of the NGOs is needed.

## Competing interests

The author(s) declare that they have no competing interests.

## Authors' contributions

ES designed, planned and carried out the study, wrote the manuscript and interpreted the data. LK participated in the design of the study and helped draft the manuscript. Both authors read and approved the final manuscript.
